# Case Series of Clinical Findings of Multi-System Inflammatory Syndrome in Children in Contrast to Kawasaki Disease

**DOI:** 10.7759/cureus.16446

**Published:** 2021-07-17

**Authors:** Htay H Aung, Oksana Nulman, Iram Nadroo, Manoj Chhabra

**Affiliations:** 1 Pediatric Medicine, New York-Presbyterian Brooklyn Methodist Hospital, Brooklyn, USA

**Keywords:** covid-19, kawasaki disease, sars-cov-2, pediatric multisystem inflammatory disease, covid-19 related, pediatric, coronavirus

## Abstract

Several months into the coronavirus disease 2019 (COVID-19) pandemic, there is growing concern over an increase in the incidence of severe acute respiratory syndrome coronavirus 2 (SARS-CoV-2)-linked Kawasaki-like disease in the pediatric population. The pediatric patients presented to the emergency room with impending shock in the setting of an atypical Kawasaki picture. On May 14, 2020, the CDC Health Alert Network released a case definition for this evolving syndrome and named it multi-system inflammatory syndrome in children (MIS-C). We report three cases of MIS-C associated with SAR-COV2 who presented to our emergency room. Persistent fever was present in all three patients and mucocutaneous and gastrointestinal symptoms were the most common associations. All three patients were found to have antibodies to COVID-19. MIS-C is a similar but distinct entity as compared to Kawasaki disease. High inflammatory markers are supportive of the diagnosis, and cardiac evaluation is crucial in MIS-C. High suspicion for the diagnosis and low threshold for workup will prevent delayed treatment.

## Introduction

While the rate of coronavirus disease 2019 (COVID-19) infections in adults appears to be decreasing, there is growing concern over an increase in the incidence of Kawasaki-like disease linked to severe acute respiratory syndrome coronavirus 2 (SARS-CoV-2) infection in the pediatric population [1]. We are learning about the clinical manifestations of COVID-19 in children, some of whom are asymptomatic while others have a fever with respiratory and gastrointestinal symptoms. These symptoms usually appear to be mild and tend to self-resolve without any apparent complications [2]. However, a small subset of pediatric patients who were exposed to SARS-CoV-2 or contracted COVID-19 are presenting with Kawasaki-like features, complicated by multi-system involvement, including the cardiac system [3].

The CDC announced a case definition for multi-system inflammatory syndrome in children (MIS-C) on May 14, 2020. The timing of MIS-C suggests an immunological phenomenon similar to Kawasaki disease (KD) [4]. KD is an acute self-limiting vasculitis with a predilection for the coronary arteries that affects previously healthy children [5]. Echocardiography is indispensable in diagnosing coronary artery vasculitis and monitoring its complications.

It is still unclear whether MIS-C has a predilection for coronary arteries like KD does. According to recently published articles, in cases of SARS-CoV2-linked MIS-C, baseline EKGs were non-specific and the most common echocardiographic finding was ventricular dysfunction, which could lead to shock [6]. It has become increasingly clear that SARS-CoV2-linked MIS-C can be a serious condition, requiring immediate life-saving intervention [7]. In this review, we present the clinical manifestations and various echocardiographic findings associated with MIS-C in our patients, with the goal of identifying both common and specific findings, thereby helping clinicians identify and manage this newly evolving syndrome.

## Case presentation

Case 1

A previously healthy five-year-old male presented to our emergency department (ED) with the chief complaint of five days of fever [maximum temperature (Tmax) of 40°C] associated with vomiting, sporadic watery diarrhea, resolving red puffy eyes, and rashes over his left lower leg. His parents also reported a mild cough during the first few days of illness. They noticed fast and noisy breathing on day five of illness, and this prompted them to bring the patient into the ED. Two weeks prior to the onset of symptoms, the patient’s father was noted to be SARS-CoV-2 positive. On physical examination, the patient was alert and well-appearing but febrile at 38.1°C, tachycardic at 162 bpm, and mildly tachypneic at 30 bpm without any chest wall retractions. His blood pressure was within normal limits. He was also noted to have significant bilateral conjunctivitis as well as a small oral ulcer.

**Figure 1:**
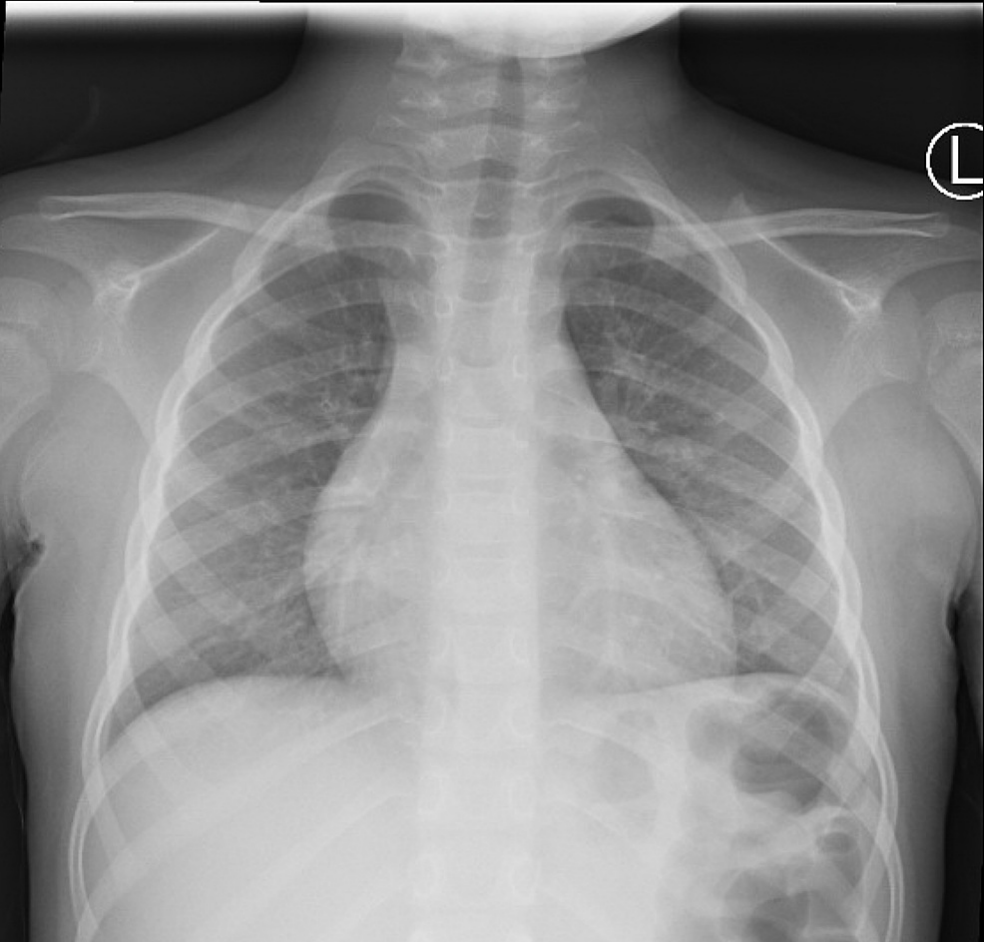
CXR showing streaky perihilar airspace opacity and mild peribronchial thickening

His complete blood count (CBC) was significant for anemia with a hemoglobin of 8.9 mg/dL; white blood cell (WBC) count was at the upper limit of normal with associated neutrophilia and lymphopenia; and normal platelets. Liver enzymes and serum creatinine were within normal limits but there was hyponatremia (Na 130 mmol/L) and hypoalbuminemia (albumin 3.1 g/dl). Inflammatory markers erythrocyte sedimentation rate (ESR) and C-reactive protein (CRP) were elevated at 123 mm/hr and 322 mg/L. Given persistent tachycardia, Troponin I levels were obtained and were noted to be high as well (.081 ng/ml). His blood cultures remained negative. While he was noted to be negative for SARS-COV-2 reverse transcription-polymerase chain reaction (RT-PCR), he was positive on antibody testing. Chest X-ray (CXR) revealed streaky perihilar airspace opacity and mild peribronchial thickening, but no focal consolidations (Figure 1). The cardiac shadow was normal on CXR.

**Figure 2:**
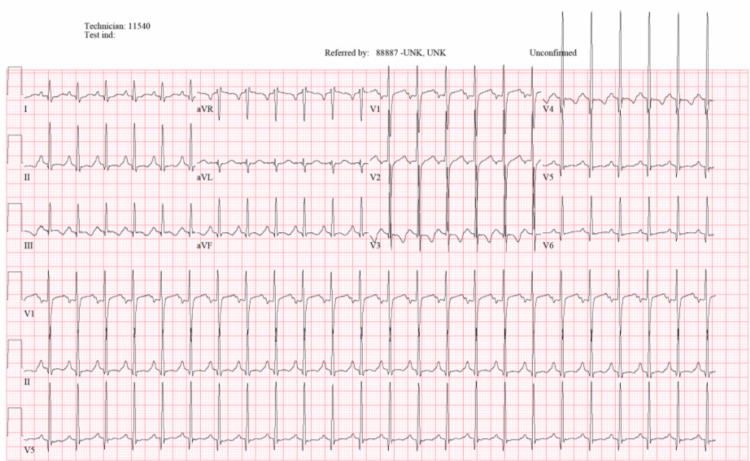
EKG showing sinus tachycardia, right atrium enlargement, and ST/T wave changes

EKG and echocardiogram (Echo) revealed non-specific abnormal findings. EKG showed sinus tachycardia, right atrium enlargement, and ST/T wave changes (Figure 2).

**Figure 3:**
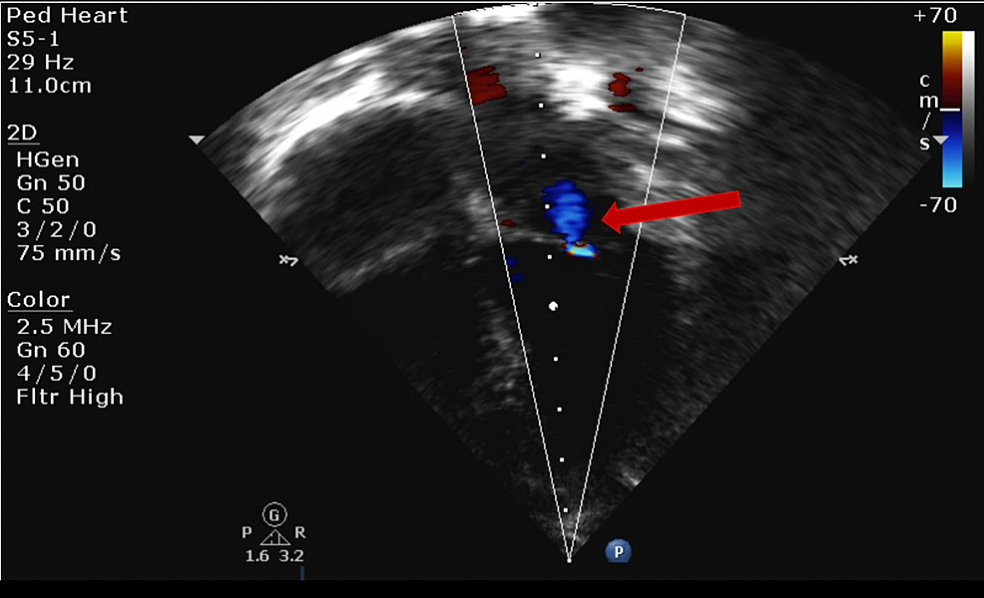
Transthoracic echocardiogram (TTE) apical four-chamber view showing mild mitral regurgitation

**Figure 4:**
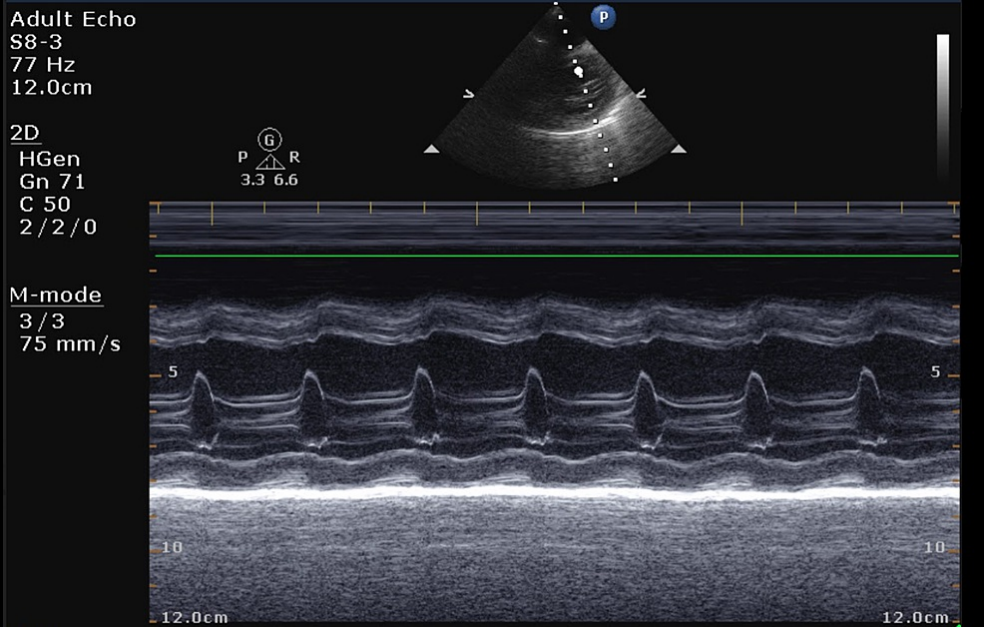
Transthoracic echocardiogram (TTE) parasternal short-axis M mode showing decreased left ventricular systolic function

Echo showed mild mitral regurgitation (MR) and tricuspid regurgitation (TR) with poor cardiac function (ejection fraction [EF] of 21%) (Figures 3-4). The remainder of the cardiac anatomy, including the coronary arteries, was unremarkable.

Case 2

A 15-year-old female with significant past medical history (PMH) of poorly controlled asthma presented to the ED with four days of fever (Tmax 38.9°C) with intermittent frontal headache, pleuritic central chest pain, and epigastric pain. She denied any respiratory symptoms. There were no known sick contacts. In the ED, she was febrile at 38.4°C and tachycardic at 136 bpm. The remainder of her vital signs were within normal limits. She was noted to have bilateral conjunctivitis and macular palmar rashes. Aside from the tachycardia, her cardiac, chest, and abdominal examinations were within normal limits.

**Figure 5:**
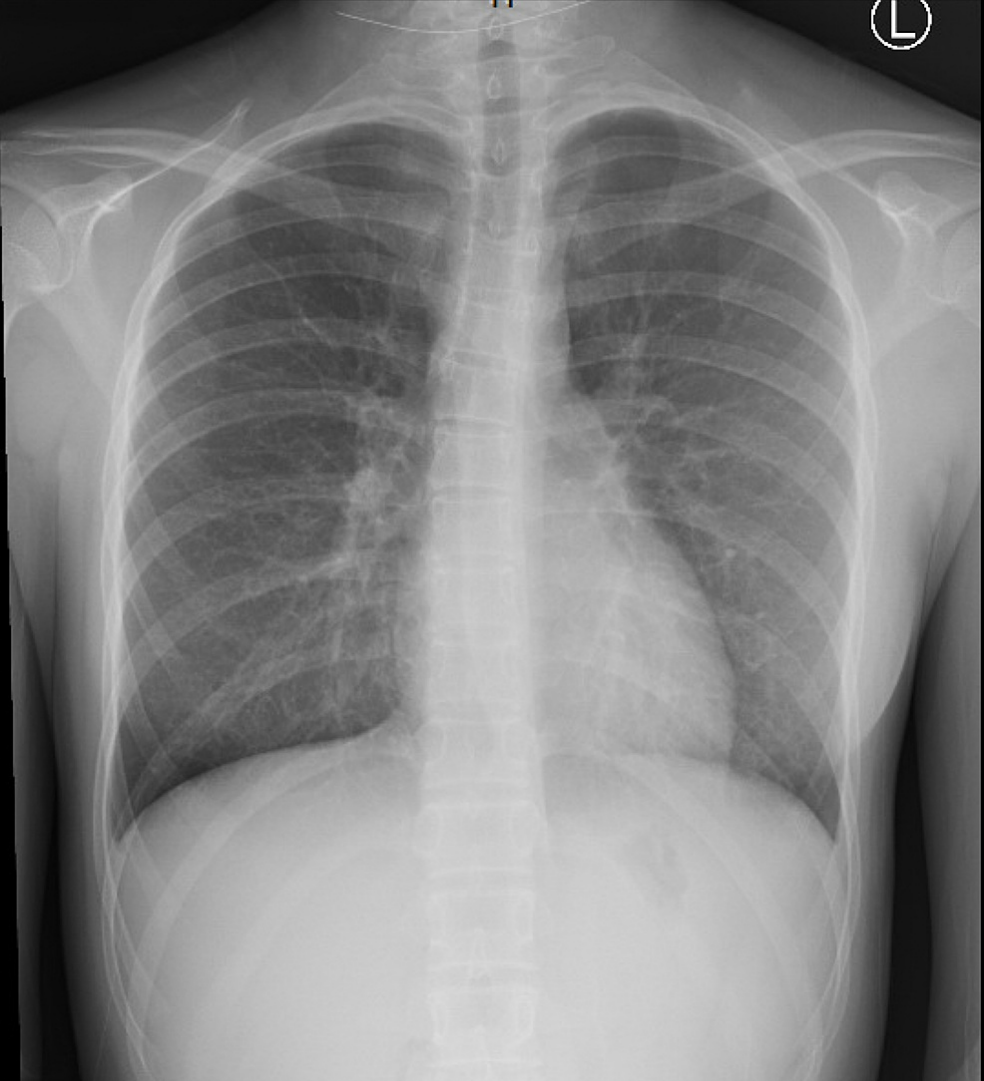
CXR showing mild hyperinflation with prominent interstitial markings CXR: chest X-ray

CBC was unremarkable except for neutrophilia and lymphopenia. Liver function tests, renal function tests, and electrolytes were within the normal limit. CRP and ferritin levels were elevated at 216 mg/L and 340 ng/ml, respectively, as were fibrinogen and D-dimer levels at >700 mg/dl and 1423 ng/ml, respectively. Pro B-type natriuretic peptide (Pro-BNP) was also found to be significantly elevated at 6000s while other cardiac markers, such as Troponin-I and CK-MB, were normal. Blood culture and urine culture remained negative. This patient was also noted to be SARSCoV2 negative on PCR but had a positive antibody test. CXR revealed mild hyperinflation with prominent interstitial markings (Figure 5).

**Figure 6:**
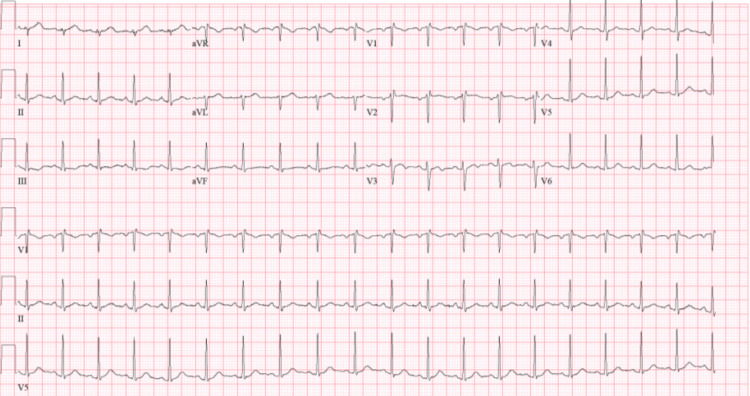
EKG showing sinus tachycardia

**Figure 7:**
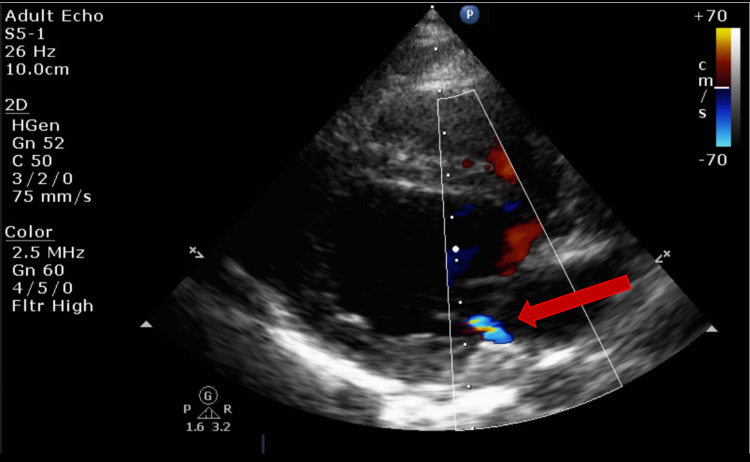
Transthoracic echocardiogram (TTE) parasternal long-axis view showing mild mitral regurgitation

EKG showed sinus tachycardia and echo findings were significant for mild MR, mild pericardial effusion, and thickening of left ventricular (LV) posterior wall with poor function (EF 23-25%) (Figures 6-7). Coronaries were noted to be normal.

Case 3

A six-year-old male, with no PMH, presented with fever for six days (Tmax 39.6°C), associated with generalized abdominal pain, followed by vomiting, facial rash, and itchy eyes. The parents also reported a mild cough and some rhinorrhea. The patient’s uncle was noted to be SARs-CoV-2 positive two months ago. The patient’s vital signs were within normal limits on presentation except for tachycardia at 123 bpm with no associated fever. On physical examination, he was noted to be well-appearing, with bilateral conjunctivitis, chapped lips, and generalized fine papular rash. Chest and abdomen examinations were unremarkable.

**Figure 8:**
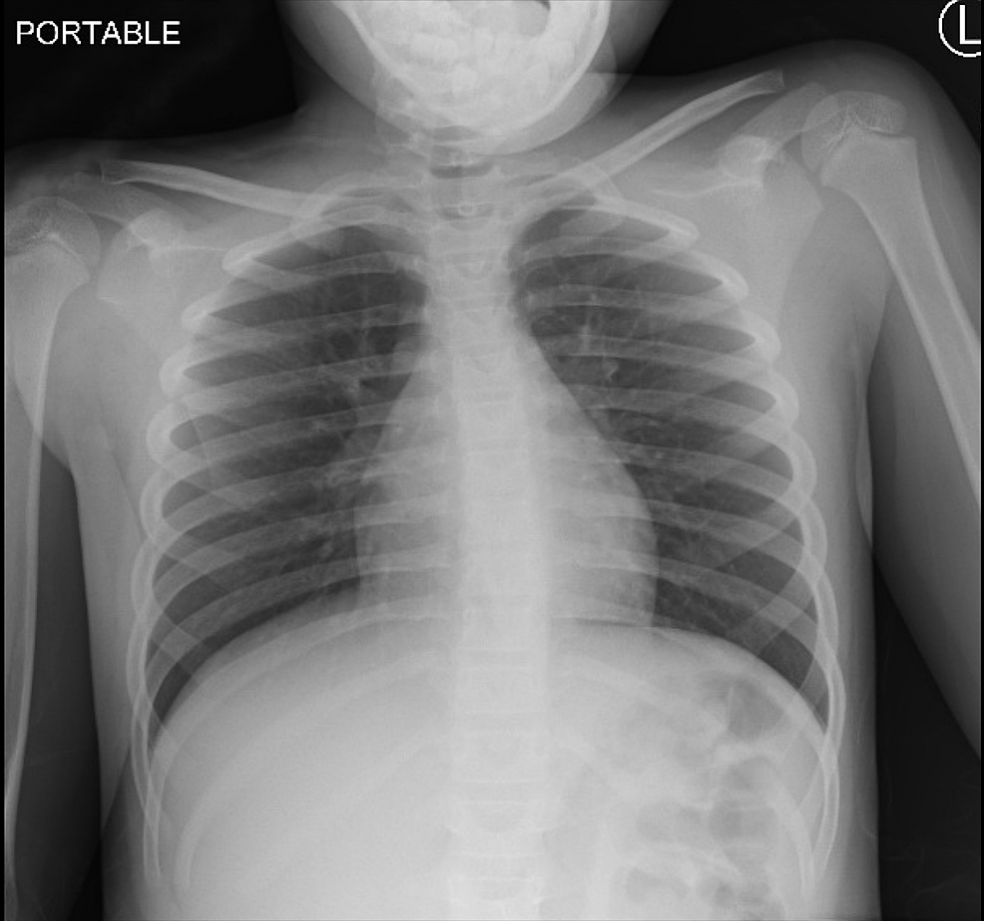
Normal CXR CXR: chest X-ray

**Figure 9:**
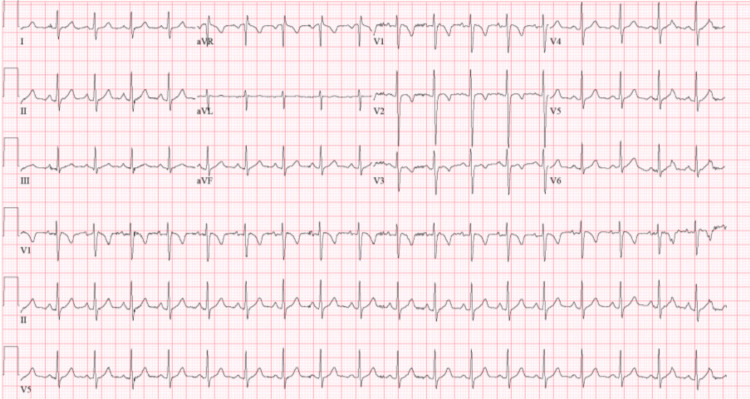
EKG showing sinus tachycardia

CBC, basic metabolic panel (BMP), and liver function tests were within normal limits. CRP, ESR, and D-dimer were increased (CRP 23.3 mg/dl, ESR 57 mm/hr, D-dimer 341.5 ng/ml) but other inflammatory markers (ferritin, lactate dehydrogenase (LDH), and procalcitonin) and cardiac markers (CK-MB, Troponin I, and Pro BNP) were normal. Blood culture, urine culture, and throat culture remained negative. CXR and EKG were unremarkable (Figures 8-9).

**Figure 10:**
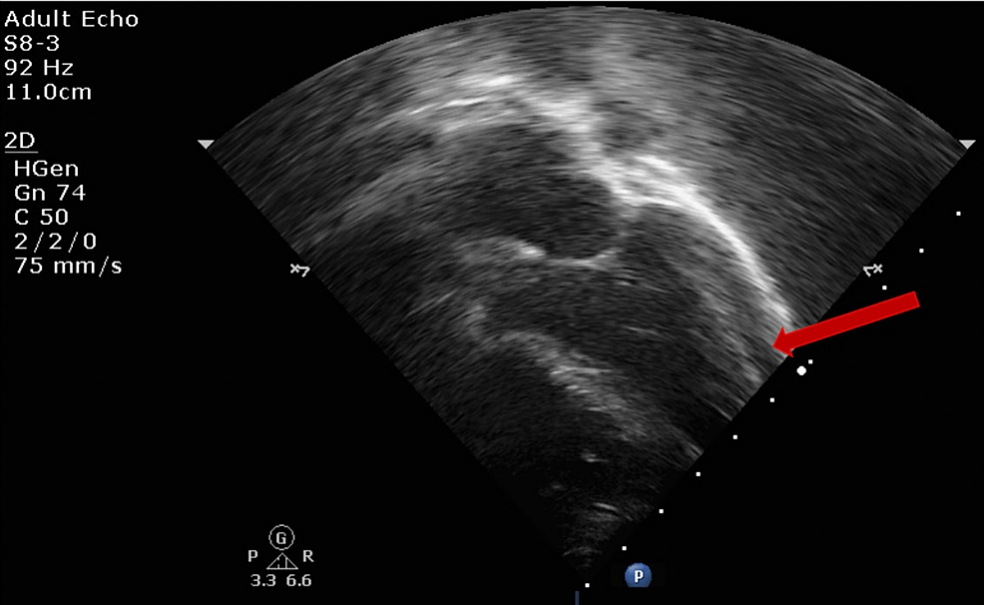
Transthoracic echocardiogram (TTE) subxiphoid short-axis view showing trivial pericardial effusion

**Figure 11:**
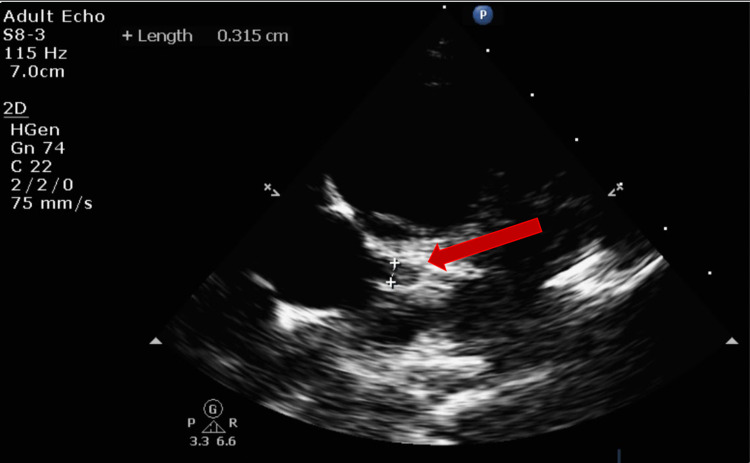
Transthoracic echocardiogram (TTE) showing left coronary prominence

However, a trivial pericardial effusion and prominent left coronary were appreciated on echo (Figures 10-11).

EF was noted to be 69%, and the remainder of the cardiac anatomy was noted to be within normal limits. This patient was also noted to be SARS-CoV-2 negative on PCR but had a positive antibody test.

## Discussion

At the beginning of the COVID-19 pandemic, we thought that pediatric patients were less vulnerable to the virus as compared to adults [2]. Several months into the pandemic, we started seeing a newly evolving inflammatory syndrome in pediatric patients who were exposed to SARS-CoV-2. The New York State Department of Health has issued a statement that as of May 5, 2020, “sixty-four suspected pediatric clinical cases compatible with multi-system inflammatory syndrome associated with COVID-19 have been reported in children in New York State hospitals [8].

On May 14, 2020, the CDC Health Alert Network released a case definition for MIS-C [9]. The health advisory provided the following criteria, all of which need to be met to qualify as MIS-C: an individual younger than 21 years; presenting with fever; ≥38°C for ≥24 hours, or report of subjective fever lasting ≥24 hours; elevated inflammatory markers; clinically severe illness requiring hospitalization, with multiorgan (≥2) involvement; patients should not have an alternative diagnosis to explain their illness; patients should be positive for current or recent SARS-CoV-2 infection by RT-PCR, serology, or antigen test; or COVID-19 exposure within the four weeks before the onset of symptoms.

As of March 1, 2021, there was a total of 2617 MIS-C cases meeting the above criteria and 33 MIS-C deaths meeting the case definition. The question of why some children develop MIS-C after COVID-19 or after contact with someone who has COVID-19 and some do not remains unanswered [10].

We reported three cases of MIS-C. Persistent fever was seen in all three patients. Fever is commonly associated with mucocutaneous manifestation such as various forms of rashes, conjunctivitis, and oral mucosa ulcer. While the fever and mucocutaneous findings in MIS-C are similar to those of Kawasaki disease, gastrointestinal symptoms, such as anorexia, nausea, vomiting, diarrhea, and abdominal pain, favor the diagnosis more toward MIS-C than KD. Furthermore, respiratory symptoms that are commonly seen in adults are less prominent in children.

Inflammatory markers, including, ESR, CRP, ferritin, fibrinogen, and D-dimer, were high in all three patients (Table 1). Cardiac markers, such as Troponin I and BNP, were significantly high only in Patient 1.

Patient 1 has been exposed to SARS-CoV-2 2 weeks prior to presentation and Patient 3 had confirmed exposure two months prior to presentation. All of them were noted to be SARS-CoV-2 negative on PCR but had a positive antibody test, which proposed a possible immune-mediated inflammatory response to the COVID-19 virus.

Common abnormal vital signs seen in most patients, apart from fever, were tachycardia with borderline blood pressure. Fluids have to be cautiously given due to the possible association of viral-induced myocarditis. That is when cardiac imaging and lab markers come into play in management.

Echocardiogram findings were variable in all three patients, including impaired ventricular function, mild/trivial pericardial effusion, prominent coronary arteries, and mild functional heart valve abnormalities such as mitral regurgitation and tricuspid regurgitation. Unlike KD, abnormal echo findings were seen early in the course of the disease in MIS-C and severe cardiac involvement like ventricular dysfunction can lead to death if left untreated or delay in treatment. All three patients were admitted to the in-patient unit for close cardiopulmonary monitoring due to the concern for rapid hemodynamic decompensation and treated with IVIG (IV immunoglobulin). Echo(es) were repeated before discharge and during the outpatient follow-up and all abnormal findings were resolved.

Ever since the first patient, physicians in our institution were setting a low threshold for workup of MIS-C in pediatric patients who come in with persistent fever, various rashes, and gastrointestinal (GI) symptoms in order to diagnose and treat early. Workup includes basic labs, inflammatory markers, cardiac markers, SARS-COV-2 PCR, and antibody testing (Table 1). An echocardiogram should be performed as soon as available; findings are variable but poor ventricular function and pericardial effusion are common. Coronary artery prominences are noted as in KD as well.

**Table 1:** Comparison of the laboratory values of the presented cases BUN: blood urea nitrogen; AST: aspartate aminotransferase; ALT: alanine transaminase; ESR: erythrocyte sedimentation rate; CRP: C-reactive protein; PT: prothrombin time; INR: international normalized ratio; aPTT: activated partial thromboplastin time; Pro-BNP: pro-B-type natriuretic peptide; LDH: lactate dehydrogenase; SARS-COV-2: severe acute respiratory syndrome coronavirus 2; RT-PCR: reverse transcription-polymerase chain reaction

	Patient 1	Patient 2	Patient 3
CBC			
Hb (g/dl)	8.9	13.9	11.6
WBC With differential (K/µL)	15.2 With Neutrophils – 85.2%, Lymphocytes – 9.2%	11.9 With Neutrophils – 92.4%, Lymphocytes – 3%	5.7 With Neutrophils – 60.6%, Lymphocytes – 31.5 %
Platelets (K/µL)	233	213	268
Renal Function With Electrolytes			
BUN (mg/dl)	14	15	11
Creatinine (mg/dl)	0.42	0.62	0.51
Na+ (mmol/L)	130	135	139
Other Electrolytes	Normal	Normal	Normal
Liver Function Test With Proteins			
AST (unit/L)	33	29	29
c (unit/L)	56	38	34
Total Protein (g/dl)	7.3	6.7	7.8
Albumin (g/dl) [reference: 3.4-5]	3.1	3.3	3.6
Inflammatory Markers			
ESR (mm/HR) [reference: 3-13]	123	-	57
CRP (mg/L) [reference: =< 3]	322	216	23.3
Ferritin (ng/ml) [reference: 8-252]	865.6	340.8	46.8
Coagulation Studies			
PT (INR) (sec) [reference: 9.6-12.7]	14.4/1.1	17.1/1.4	13.3/1.16
aPTT (sec) [reference: 25-35.6]	30.5	38.5	32.9
Fibrinogen (mg/dl) [reference: 263-623]	598	>700	546
D-dimer (ng/ml) [reference: 30-230]	4350	1423	341.5
Cardiac Markers			
Troponin I (ng/ml) [reference: =< .045	.081	.015	.019
Pro-BNP (pg/ml) [reference: =< 125]	27768	6278	78
LDH (unit/L) [reference: 84-246]	266	356	210
SARS-COV-2 Assays			
SARS-COV-2 RT-PCR (Nasopharyngeal Swab)	Negative	Negative	Negative
Antibody Serology	Positive	Positive	Positive
Microbiology			
Blood Culture	Negative	-	Negative
Urine Culture	-		Negative

## Conclusions

In conclusion, MIS-C is a similar but distinct entity as compared to KD. A history of exposure to COVID-19 is important although not universally available. Persistent fever with high inflammatory markers are seen in almost all patients. However, cardiac markers, such as BNP, cannot be relied on as the marker of this syndrome. The echocardiogram findings are variable but focus on ventricular function and prominence of coronary arteries. Mild pericardial effusion is a non-specific but common finding in MIS-C. A multidisciplinary approach, including a cardiologist, intensivist, and infectious disease specialist, should be involved in individualized management. We hope our cases contribute to the literature pool. Ultimately, a complete understanding of the pathophysiology and evidence-based management guidelines awaits further studies.
